# Exploring social media adoption by nurses for nursing practice in rural Volta, Ghana

**DOI:** 10.1002/nop2.1685

**Published:** 2023-02-25

**Authors:** Nathan Gamor, Gladys Dzansi, Kennedy Dodam Konlan, Eliasu Abdulai

**Affiliations:** ^1^ Catholic Hospital Battor Battor Volta Region Ghana; ^2^ School of Nursing and Midwifery University of Ghana Legon Ghana

**Keywords:** attitude towards, behavioural intention, nurse, nursing practice, perceived ease, perceived usefulness, social media

## Abstract

**Aim:**

The purpose of the study was to inquire into social media adoption by nurses for nursing practice.

**Design:**

An exploratory descriptive qualitative design was employed in understanding social media adoption for nursing care among nurses.

**Method:**

A purposive sampling technique was employed to recruit 12 participants for the study. A semi‐structured interview guide was used to conduct in‐depth interviews which were audiotaped, transcribed verbatim, coded and analysed. Thematic analysis was used to analyse the data with NVivo 12.

**Results:**

The findings revealed nurses found social media to be useful for the dissemination, and reception of information, professional development and enhanced referral networks. Apart from its usefulness, participants believe that it is easy to navigate its apps, clear and understandable to use and does not involve much mental effort hence their favourable attitude towards use. Some participants also believe that inaccurate information, privacy and confidentiality concerns, distraction and addiction were some potential risks that are associated with its usage in nursing practice. Due to this, some participants developed a negative attitude towards its usage.

**Patient or Public Contribution:**

Twelve nurses actively participated in the study.

## 1 BACKGROUND TO THE STUDY

Globally, the internet is the backbone of our society with about 4.66 billion people globally using the internet today representing roughly 59.9% of the world's population (Kemp, 2021). Chaffey (2021) and Kemp (2021) also noted that more than half of the world's population is using social media, with the average user having almost nine accounts on different networking sites.

As a result of social media penetration worldwide, its user base has become more reflective of the general population, as more people have embraced social media internationally (Dwivedi et al., 2021). Young adults were among the first adopters of social media and continue to use these platforms at high levels, although in recent years, the use by older adults has increased (Koiranen et al., 2020).

Social media are interactive media technologies that enable the creation and sharing of information, ideas, interests and other forms of expression via virtual communities and networks (Aichner et al., 2021). While there are challenges to the definition of social media due to the variety of stand‐alone and built‐in social media services currently available, some common features include interactive Web 2.0 Internet‐based applications, user‐generated content, such as text posts or comments, digital photos or videos and data generated through all online interactions, and users create service‐specific profiles for the website or app that are designed and maintained by the service provider (Aichner et al., 2021). For the purpose of this study, Social media is operationally defined as networking or interacting with other people to share information via an electronic communication medium such as Facebook, WhatsApp, Snapchat, telegram, YouTube, etc.

Social networking platforms provide the individual user with several characteristics that serve various purposes (Kircaburun et al., 2020). There are hundreds of social media sites and platforms in the world. Some include Facebook, WhatsApp, Twitter, Instagram, Telegram, Snapchat, Youtube and Google+ are the most popular and widely recognized social media sites in the world (Alexa, 2021). Lefebvre et al. (2020) also observed that Facebook, WhatsApp, Twitter, Instagram, Snapchat, YouTube and Google+ as the most frequent social media sites or apps used by nurses. These apps are used to interact with family members and friends, colleagues, meet new people, share interests and, most importantly, deepen understanding and awareness of different subjects and thus enhance the learning experience (Kapoor et al., 2018).

Hospitals and healthcare professionals' use of social media have expanded dramatically. This is shifting the nature and pace of the healthcare partnership between individuals and health organizations. The general public, patients and health professionals use social media to interact about health problems (Gupta et al., 2022). Works of literature (Ahad et al., 2019; Khezr et al., 2019; Kross et al., 2021) show that social media has significant potential value for this wide area of emerging healthcare technology as they provide a new way of accessing and exchanging information, encouraging social collaboration and stakeholder participation, and improving contact between people and allowing users to directly engage in health‐related issues of concern (Stellefson et al., 2020).

In Africa, doubts have been raised about the general benefits of internet access and social media use. But in recent years, Africans actively using social media stood at 216 million (representing 17% penetration), and out of this number 202 million access social media on their mobile phone (Kemp, 2021). Hence, healthcare industries in Africa are also gradually adopting social media in their service delivery as a result of escalating competition and patient demand for the highest quality care (Adetunji & Moses, 2022).

While social media technology has the potential to improve nursing care, it is not without risks. Privacy and confidentiality, unprofessional behaviour and organizational risk are risks‐related issues that nurses in Africa face with the use of social media technology while at work (Khan et al., 2021). Understanding what influences nurses to adopt social media technology in nursing care will help to ensure that the nurses' focus on patient care is never lost as risks involved in using social media are being avoided (Khan et al., 2021).

It is yet too early to tell whether or not social media will have a positive or negative impact on the healthcare industry, but early research suggests that it will have a positive impact on patient care. It is possible to enhance information about the appropriate integration of social media in health care by studying the traits, attitudes and external factors of users who contribute to the usage purpose and frequency of use of social media. It is therefore a challenge for this study to focus on what motivates nurses to use social media in their daily work.

There is enough evidence to suggest that the majority of nurses in Ghana are adopting social media to engage in the promotion of health, patient education, outreach, professional development and other nursing‐related activities. But little has been done in terms of evaluating the factors that impact the adoption of social media by Ghanaian nurses. As a result, it is necessary to comprehend the factors that fuel the adoption of social media by nurses at Catholic Hospital Battor in the Volta Region. The study's purpose is to answer the following questions: How do nurses perceive social media to be useful in nursing practice? How do nurses perceive the ease with which social media can be used to improve nursing practice? What is nurses' perception of the use of social media in nursing practice?

## 2 THEORETICAL FRAMEWORK

Fred Davis' Technology Acceptance Model (TAM) was employed as the underpinning model, taking into account the study's goals. Because of its structure, TAM is ideal for simulating how users will accept new information systems or technologies. To motivate a user to accept new technology, TAM uses core variables such as perceived utility, perceived ease of use and attitudes towards new technology. Outcome variables are then used to measure whether or not a user has accepted the technology. One way or another, perceived usefulness (PU) and perceived ease of use (PEU) explain the results.

### 2.1 Perceived usefulness of social media

According to the TAM, perceived usefulness and ease of use of technology determine attitude and usage intention, which leads to acceptance and usage of the given technology. Perceived usefulness is defined as an individual's belief that using new technology will improve or enhance his or her performance (Davis, 1989). In another word, it is the benefits or the advantages the user derives from the use of the technology.

In the context of nurses' social media adoption for nursing practice enhancement, usefulness refers to the extent to which nurses feel that using social media would improve their nursing care or productivity, hence improving the health institution's outcome. A technology system is highly perceived as useful if the user believes that its existence will positively enhance the user practices hence the user perceives the system to be an effective way of practicing the tasks (Henderson et al., 2017).

### 2.2 Perceived ease of use of social media

The degree to which a person believes that using a system will be simple is characterized by perceived ease of use (Davis, 1989). Perceived ease of use of social media in the context of this study refers to the extent to which users believe that using social media to improve performance is effortless. Furthermore, because the human effort is a finite resource, users are more likely to adopt an application that is seen to be easier to use than another (Davis, 1989). The point of view of the user on the ease of understanding greatly aids to determine the potency and the degree of the user's adaption to new technology (Davis, 1989). Dzandu et al. (2016) discovered that if a system is very simple to use, people will go to great lengths to learn about its capabilities and eventually desire to keep using it.

### 2.3 Attitude towards using social media

Attitude towards the usage of technology refers to a physical propensity that is shown by appraising a specific entity favourably or unfavourably (Davis, 1989). According to TAM, personal attitudes about technology influence the adoption and use of that technology (Bogea & Brito, 2018). Similarly, attitude towards social media use has a direct effect on user intention (Rolls et al., 2016). Al‐Shdayfat (2018) revealed in a study on undergraduate student nurses' attitudes towards using social media websites that the majority of nursing students have good attitudes about social media use for educational and professional objectives, even though just a minority use it for professional purposes. Oducado et al. (2019) also stated that nurses had a favourable attitude and impression of using social media properly for professional purposes.

## 3 METHODS

### 3.1 Design

An exploratory descriptive qualitative design was employed in understanding social media adoption for nursing care among nurses. Situating the in‐depth exploration of this phenomenon in the technology adoption model; the usefulness, ease of use, attitude and behaviour towards social media use was explored. This approach allowed the researchers to gain an in‐depth understanding of factors that influence social media adoption by nurses.

The constructivist philosophical paradigm underpinned the methodological approach of this study which is the position the researchers took. According to this philosophical paradigm, people develop their understanding and knowledge of the universe by experiencing the world and reflecting on those experiences (Kivunja & Kuyini, 2017). It is based on the analogy or basis that people form or construct much of what they learn through experience.

The researchers believe nurses' experience with social media usage in their nursing practices is a phenomenon with several meanings and interpretations. As a result, the constructivist paradigm, which underpins qualitative inquiry, was employed. As a result, an exploratory qualitative approach was suited for the study because it allowed participants to express themselves freely and honestly.

### 3.2 Study setting

The study was conducted at a rural district hospital in the Volta Region of Ghana. The hospital is located in Battor the district capital of North Tongu District. It is among the deprived districts of Ghana. The population of the district is 89,777 of which 42,492 are male (representing 47.3%) with 47 females and 285 representing 52.7% (Ghana Statistical Service, 2013). According to Ghana Statistical Service (2013), 37.3% of the district's population aged 12 and up own a mobile phone. In the District, 2.2% of the population aged 12 and up uses the Internet. Furthermore, the survey shows that 444 houses have desktop/laptop computers, accounting for 2.4% of the district's total households.

### 3.3 Sampling technique and sample size

A purposive sample technique, also known as a judgmental sampling technique, was used to elicit a wide range of opinions from participants. Participants were purposefully picked because they might provide useful insights into the understanding of social media use and adoption. Wards and other unit managers were contacted to help identify nurse participants who fall in the inclusion criteria and can provide relevant perspectives on how social media are used. A total of 12 nurses and midwives were recruited relative to information saturation (Polit & Beck, 2013).

### 3.4 Data collection procedures

A semi‐structured interview guide based on the study objectives and TAM was used to collect the data. All the interviews were conducted in English after ethical clearance. The place and time of the interviews were at the convenience of the participants. Interviews lasted between 30 and 45 min apiece. The interview began with major and probing questions from the researchers. This provided enough foundation for the participants to make extensive and convincing responses. Other follow‐up questions aimed to elicit more information about the participants' experiences with social media were asked. Other probing questions were used to bring the participants back on track when they lost track of the question. The interviews were audio‐recorded with a digital voice recorder with the consent of participants. The field notes on the context and non‐verbal behaviour during the interviews were written together with the verbatim transcriptions of the interviews. Field notes included reflections during data collection to ensure that nurses' perspectives were accurately recorded.

### 3.5 Data management and analysis

Data were analysed using thematic analysis with NVivo software. The researchers transcribed the recorded interview verbatim into a personal computer at the end of each day of the interview, taking into consideration the field notes. The transcripts were matched to the audio recordings and missing connections were filled in to guarantee that the transcripts were accurate (Nowell et al., 2017). The researchers were given copies of the transcripts to read and comment on. The actual analysis entailed reading and re‐reading the transcripts to detect common sentences or concepts, which were then differentiated by assigning codes. Based on their similarities, these codes were divided into subgroups. Subcategories with comparable meanings were put together into categories, which were then further divided into themes (Braun & Clarke, 2006). The themes were modified several times until they were suitable for presenting the findings per the study's aims.

### 3.6 Ethical considerations

Before carrying out the study, the researchers sought Research Ethics Committee approval from the Institutional Review Board (IRB) of the Christian Health Association of Ghana with IRB Number CHAG‐IRB PIN: GHAG‐IRB01012021. This approval letter was sent to the management of the Catholic Hospital Battor and the District Director of Health Services, North Tongu District, to obtain permission before the nurses and midwives were recruited for the study. Ethical principles of research involving human participants such as anonymity, respect for human dignity, well‐being and justice (Polit & Beck, 2013) have been adhered to.

### 3.7 Methodological rigour

In qualitative research, methodological rigour investigates the degree to which the research results truly reflect the participants' experience or state of being extremely precise, meticulous or precise or the quality of being thorough and accurate (Cypress, 2017). Guba and Lincoin (1982) offered four sets of standards that can be used for this purpose to improve the trustworthiness of a sample. These include credibility, dependability, confirmability and transferability.

Several procedures were used to keep trustworthiness. For instance, one researcher gathered all the data, ensuring that the same questioning strategies were applied. Also, to establish credibility in this study, the researchers made sure that participants met the inclusion criteria, pre‐test of the interview guide, which helped to adjust some of the questions, also contributed to the research's credibility. The concurrent data analysis also helped ensure that the themes in this study were fully developed. Apart from these, the researchers kept an audit trail of field notes, audio recordings, analytical notes and coding details. A personal notebook was also kept, and any motivations, preferences and assumptions that might impact the research process were recorded.

In the spirit of self‐reflection, we acknowledge our position as researchers who are not active social media users before presenting the findings. The lead researcher works in the research areas and has a negative attitude towards using social media at work. Others, even though are not active social media users, are interested in the use of technology in nursing. The first and last authors are both novice researchers. We acknowledge that our perspective influenced this study to some extent.

## 4 RESULTS

### 4.1 Demographic characteristics

Twelves nurses between 23 and 43 years old participated in the study. The nurses included eight females and four males. The nurses had worked for a period ranging from 1 to 11 years. Their ranks ranged from Staff nurses/midwives to Principal nursing/Midwifery officers. These demographic characteristics of participants can be seen in Table 1.

**TABLE 1:** Participants' characteristics profile

Pseudonym	Sex	Age	Educational level	Nursing grade	Years in service
BN001	Female	30	Diploma	Senior Staff midwife	6
BN002	Female	32	Degree	Midwifery Officer	8
BN003	Male	35	Degree	Nursing Officer	4
BN004	Female	33	Master	Senior Nursing Officer	11
BN005	Male	30	Degree	Senior Nursing Officer	7
BN006	Male	36	Degree	Nursing Officer	7
BN007	Female	28	Degree	Staff Nurse	3
BN008	Female	34	Degree	Senior Nursing Officer	8
BN009	Female	31	Master	Senior Nursing Officer	8
BN010	Male	34	Degree	Nursing Officer	10
BN011	Female	23	Degree	Nursing Officer	1
BN012	Female	43	Degree	Senior Nursing Officer	8

The study generated four main themes: Nurses' perceived usefulness of social media, Nurses' perceived ease of use of social media, Perceived Potential Risks of Use and attitude towards the use of social media. Table 2 shows the four Themes and the 12 Sub‐themes generated from the thematic analysis.

**TABLE 2:** Themes and sub‐themes

Major themes	Sub‐themes
Nurses' perceived usefulness of social media	Dissemination and Reception of Information
	Professional Development
	Enhance Referral Network
	Clearer and Understandable
Nurses' perceived ease of use of social media	Low Mental Effort (Stress‐Free)
	Easy to Navigate
	Inaccurate Information
Perceived potential risks of use	Privacy and Confidentiality Concerns
	Distraction
	Addiction
	Favourable approach
Attitude towards the use of social media	Unfavourable Attitude

### 4.2 Nurses' perceived usefulness of social media

Perceived Usefulness is the extent to which nurses deem social media to be utile, relevant or boost their nursing practices. Nurses mentioned dissemination and reception of information, professional development and enhanced referral network as some of the usefulness of social media in nursing practice.

*……Advantages being that information is shared sometimes about current conditions, current treatment (treatment protocols), and they are shared on pages so that you can read them, no need to put them on notice board……* BN006.

In a related narration, a participant, BN011 believes that social media is far better to disseminate and receive information compare with the traditional notice board.

*Yes. As compared to notice boards, I read WhatsApp messages more often. So, when notices about programs or ward activities are posted on WhatsApp platforms, I read it much easier than when it's posted on the notice board*. BN011.

Some nurses described how social media is beneficial as a means for training, certification and education that help a nurse to succeed in their nursing career.

*……Very helpful. Of late because of this COVID, there is a whole lot of pieces of stuff that is being turned into a social media base. Even meetings, webinars, teaching, and others are being held online*. BN001.

Participants expressed their opinions that social media is useful in collaborating with patients' referral systems.

……. *Communication was mainly through referral letters and sometimes phone calls……but with social media there is a quick back and forth information transfer about the client and also the referral letters are posted on our platform*. BN008.

### 4.3 Nurses' perceived ease of use of social media

Perceived ease of use relates to the extent to which a nurse believes that making use of social media in their nursing practice would be of low mental effort or effortless, easy to use and hassle‐free. Nurses believe that social media is clear and understandable, requires low mental effort (stress‐free) and is easy to navigate.

*……. So, it is quick, easily accessible, and understandable. Everything is clear and is readily accessible. I won't say social media is so difficult to use or is a complicated technology that demands schooling before using it, is very simple to use*… BN001.

Participants described their usage experiences as stress‐free, with no critical thinking and no mental effect required to use social media for their nursing practices.

*I think it is easy. Much mental effort isn't required*. BN005 *I think it's not stressful, not nerve‐racking because using social media has become part of us. Once you can use it for your stuff, you can as well apply it to work*. BN009.

Participant believed that they can manipulate social media in any way to get nursing information for their practice.

*Well, I think it's easier. You don't need much effort to maneuver. Some of the social media types are straightforward. Some of the social media comes with self‐tutorial and it helps a lot when the going becomes tough. I think is very easy to use*. BN002.

### 4.4 Perceived potential risks of use

Perceived potential risks of use of social media described the participants perceived believed that the use of social media despite its usefulness and ease of use comes with potential risks. Inaccurate and misinformation, privacy and confidentiality concerns and distraction and addiction.

*False information is the issue here, people want to impress everybody so others might not double‐check before using them. Some people may not have done extensive research but will be in a hurry to put out information that tends to be half‐truths*. BN012.

Some participants described potential leakage of patients' confidential information as a result of the indiscriminate posting of patients' information in social media with disregard to privacy and confidentiality rules.

*……another negative impact is that we forget patients' confidentiality then take pictures of them thereby showing their faces and information to the entire world*………BN012.

Participants believe that their attention is being drawn away from their core nursing work as a result of the use of social media is a potential risk.

*The temptation of going to do other things on your phone is high. Because it's your phone, and you might see things that might draw your attention other than what you intended to do*. BN007.

Other participants stated that extreme preoccupation with social media, driven by an uncontrollable desire to use social media, and investing so much time on social media is a risk to nursing practice.

*……It speeds up the nursing practice but it can also be addictive and may delay the work unnecessarily…*. BN005.

*Most often than not, we're so much dependent on these media that every noise coming from the phone we want to check*. BN009.

### 4.5 Attitude towards using social media

Attitude towards using social media describes beliefs, feelings, values and dispositions towards the use of social media by nurses in nursing practice. Nurses depicted their negative and positive attitudes concerning social media usage. Nurses were of the view that their attitudes towards using social media were favourable and unfavourable attitude.

*It is positive and the feeling is good because I keep up with the latest trend in social media* ……BN0012.

*Negative in the sense that social media is time‐consuming. Social media per the nursing care sometimes interferes with nursing workflow. I hardly use it, especially in the ward*. BN006.

## 5 DISCUSSION

The data from the study show that nurses benefit greatly from using social media in their nursing practice. It plays a key role in information dissemination and reception. Nurses were emphatic that social media has aided in the distribution and receiving of health‐related information from colleagues in other facilities and also research findings among nurses and their patients globally, also helps in the dissemination and reception of clinical guidelines for nursing practice. This finding is consistent with other research that claims social media provides nurses with a new communication channel through which they could share and exchange health information in previously impossible ways (Farsi, 2021; Org et al., 2017). Participants were of the view that social media is more beneficial and much more useful to disseminate clinical practice guidelines (clinical protocols) for nurses than the normal traditional (notice board and books) means. This disclosure agrees with the project the European Association of Urology carried out using Twitter to disseminate clinical protocols to members (Loeb et al., 2017).

Nurses regard social media to be useful for professional development which includes training, certifications, education and discourses. Additionally, social media platforms are transforming into an accepted and financially viable means for health professionals to maintain their professional growth (Adjei et al., 2023; Maloney et al., 2017). Since best practice is constantly changing and requires nurses, doctors, researchers and educators to be lifelong learners, social media provided a platform and has evolved into a valuable technology tool for professional development.

Another finding from the study indicated that social media is very useful to enhance referral networks and communications. Participants indicated that social media is a valuable tool to strengthen the referral system in health institutions. The interactive and collaborative nature of social media tools like WhatsApp and Short Message Service (SMS) (Korsah et al., 2023) could enable nurses to communicate referral issues among themselves ahead of time. In addition, participants believed that social media content sharing ability makes it possible for patients' details in form of scans and X‐rays to be sent directly to the receiving facility ahead of time.

However, the study revealed that some participants were sceptical about social media's ability to help promptly render nursing tasks without undue delay. They believed that technical issues (poor network, information overload and slowness of device being used) that come with social media usage can slow down its use to accomplish nursing tasks. Technology can be difficult to use especially at its initial introductory stage (Vogels et al., 2020).

The current study revealed that social media is clear and understandable to use, thus its usage is free from confusion, doubt or any troubling thoughts. Invariably, clarity of social media applications is a prerequisite to understanding the use of social media apps. Nurses can therefore adopt social media apps with a good interface that is clear to understand and most appropriate to their practice.

Another revelation from nurses why they perceived social media to be easy to use is the low mental effort involved in its usage. Participants believe that its usage is stress‐free and requires minimum effort to use it to its fullest in nursing practice. This finding is consistent with Mitzner et al. (2016) and Deng et al. (2018). Their studies indicated that employees' perception that using a particular technology that will be free from physical or mental efforts will lead to its perceived ease of use hence the technology adoption.

The finding further divulges that nurses perceived social media to be easy to use if is easy to navigate its functions. This demonstrated that social media adoption is because most social media interfaces are user‐friendly, and also, it is easier to navigate the functions that allow for an easy interaction on social media platforms. This makes its application in nursing practice very simple.

The majority of the participants opined that inaccurate information on social media is a risk to nursing practice. In general, inaccurate information means incorrect, misleading or fake information. On social media, inaccurate information may also be characterized by a situation in which locating relevant information is difficult due to the less‐ordered, unauthenticated and uncontrolled nature of information created and shared (Panahi et al., 2016). The current study suggested that unauthenticated, inaccurate and misleading materials on social media are caused by the fact that communication on some of these platforms between nurses is mixed with both social and professional talks.

Furthermore, the participants revealed that the majority of this false information is disseminated by health professionals themselves through social media platforms. This probably makes patients and the general public believe whatever information that these health professionals disseminate and its consumption becomes very easy.

Another perceived potential risk of using social media in nursing that the study participants revealed are the breach of patient confidentiality and privacy. This finding is consistent with other observations in the literature (Ahmed et al., 2020; Lefebvre et al., 2020). The violation might be intentional or unintentional, and it can occur in a variety of ways (Geraghty et al., 2021). Some nurses may violate patients' privacy by posting information on social networking sites and platforms, such as posting images or videos of a patient without their authorization, commenting on patients in a derogatory manner and exposing too many patients' details, which allows them to be recognized In Ghana, these patients' privacy and confidentiality issues are incorporated into the patient charter, which is available in all health institutions throughout the country. This patients' charter specifies the privacy, rights and expectations that patients have as part of their human basic rights (Kalyuzhny et al., 2020). When their privacy is compromised, even if accidentally, patients may feel they have lost their dignity, destroying the therapeutic relationship that exists between nurses and patients (Kuruppu et al., 2022).

The study also discovered distraction or task interruption as another perceived potential risk of using social media in nursing. Participants describe distraction or task interruption as any pop‐up or buzzing sounds that result from the use of social media apps that draw away one attention from their core nursing tasks. Distractions from the social media alert have been documented to have influenced nurses and other health professionals and their decision‐making process (Reed et al., 2018). Similarly, participants noted that over‐reliance on social media while at work is a major reason for distraction that brings about task interruption. By the nature of their work, nurses are in a complicated environment that is vulnerable to distraction from social media and hence are susceptible to committing errors (Thomas et al., 2017).

Social media like any internet‐based tool can be addictive. The finding showed addiction as perceived potential risk nurses face with the use of social media in their nursing practices. Participants indicated that social media addiction is abnormal dependence and craving strongly to log on to social media apps. Participants believed that this addiction leads to investing so much time on social media which invariably interferes with nursing tasks.

The current study shows that participants have a good disposition to engage in social media usage. They believed that their favourable approach stems from the belief that social media is the best medium through which they can easily keep up‐to‐date with current issues in nursing and also update their knowledge. They held the assertion also that their attitude is positive because issues relating to the perceived risks of using social media in nursing are being upheld through policy guidelines. Notwithstanding this finding, nurses reveal unfavourable attitudes towards social media use in nursing. The opposing attitude towards social media use in nursing by the participants in this current study could probably be due to the time‐consuming nature and ethical issues that arise from its usage in nursing. This could also be that this group of participants has limited experience with social media in nursing practice coupled with technical issues like bad networks, slow speed and cost of data among others.

### 5.1 Limitations of the study

The geographical location of the study is the first restriction of this study. The study was conducted in the North Tongu district capital, Battor which is a rural setting hence the study was limited to internet networks options, unstable networks and good internet speed to work with compared with the urban setting where there are so many options, stable networks, and good internet speed. In addition, the participants were drawn from a single health facility in the area. It is possible that the findings may not apply to other hospitals in the region or the country as a whole. As a result, similar and future investigations in other facilities located in a rural setting where there are internet network options to select from, a stable network, and good speed are available are required. The outbreak of the Covid‐19 pandemic was another big stumbling block. The study took place during the global outbreak, which made data collecting a bit terrifying and difficult. This made travelling from one location to another to meet participants for the scheduled interviews challenging hence some of the interviews were through phone calls.

## 6 CONCLUSION

Social media has transformed communication technology, allowing people to use and communicate in previously unimaginable ways. This study explores social media adoption by nurses for nursing practice in rural areas of the Volta Region, Ghana. The study concluded that for each situation, the risk‐to‐benefit ratio of using social media in nursing practice should be looked at carefully. The study also reasons that the Ministry of Health should collaborate with Ghana health service, the Christian health association of Ghana and other private health agencies to develop policies that will guide the use of social media at various health institutions, especially in clinical settings. The ministry also has to work with network providers to establish good internet at healthcare institutions where there is no network, especially at rural areas.

The study concluded that for nurses to be more specific about which social media apps should be used in nursing practice, the Ghana Health Service should recommend the type of social media apps that can use by nurses in nursing practice, and also provide devices, especially to remote health institutions to enable them effectively use social media to connect with other health facilities to enhance health delivery.

On the finding that social media enhance the referral of patient to other health facilities, the study concluded that health institutions should be encouraged to embrace the use of social media to enhance the referral system. Apart from these, since nurses are already engaged in social media use in their nursing practices, authorities should formulate policies on continuous education on new trends in the use of social media in all healthcare institutions across the country. Social media has revolutionized the world, and nurses may take advantage of this transformation to benefit themselves, their patients and their profession.

## AUTHOR CONTRIBUTIONS

NG was the corresponding author and contributed to the conception, design, data analysis and manuscript writing. The manuscript was revised by GD and KDK. EA was involved in data collection, data analysis and manuscript drafting. The manuscript was read and approved by all the authors.

## FUNDING INFORMATION

This study received no funding from public, private, or non‐profit organisation. The corresponding author is not a recipient of a research scholarship.

## CONFLICT OF INTEREST STATEMENT

The authors are not connected to any organization that has a direct or indirect financial stake in the topics covered in the manuscript.

We certify that all authors listed on the title page have made substantial contributions to the work, have read the manuscript, have attested to the accuracy and legitimacy of the data and their interpretation and have given their consent for the submission of the work to the Nursing Open.

## ETHICS STATEMENT

This paper is the authors' original piece, and it has never been published or is currently being considered for publication elsewhere. The paper accurately and completely reflects the authors' own research.

## Data Availability

The data that support the findings of this study are available from the corresponding author upon reasonable request.
